# Menu item prices and promotions offered on a meal delivery app in the UK and their socio-economic patterns

**DOI:** 10.1017/S1368980025100529

**Published:** 2025-06-13

**Authors:** Yuru Huang, Nuwan Weerasinghe, Jean Adams, Holly Rippin, Kathrin Hetz, Olga Zhiteneva, Kremlin Wickramasinghe

**Affiliations:** 1 Special Initiative on NCDs and Innovation, WHO Regional Office for Europe, 2100 Copenhagen, Denmark; 2 MRC Epidemiology Unit, University of Cambridge, Box 285 Institute of Metabolic Science, Cambridge Biomedical Campus, Cambridge CB2 0QQ, UK; 3 Department of Human Ecology, University of California, Davis, Hart Hall, 301 Shields Ave, Davis, CA 95616, USA

**Keywords:** Meal delivery apps, Digital food environment, Marketing, Pricing, Promotions

## Abstract

**Objective:**

To describe menu item prices and promotions on a meal delivery app in the UK and explore their socio-economic patterns.

**Design:**

Cross-sectional descriptive analysis

**Setting:**

We analysed over 21 million menu items from 71 532 food outlets listed on JustEat across the UK. We assessed median prices and types of promotions, examining variations by cuisine (e.g. chicken dishes, pizza) and outlet type (i.e. grocery, chain takeaways). Promotions were categorised into six types: percentage off, stamp cards, free items, meal deal notifications, buy one get one free and low delivery fees.

**Results:**

The median number of food outlets accessible via JustEat was sixty-nine per postcode district with delivery access (IQR = 14–225). The median menu item price was £6·25, with small/independent takeaways showing the highest prices. Menu item prices were generally lower in more deprived areas. Promotions were prevalent, with 65·96 % of outlets offering at least one. Outlets delivering to more deprived areas tended to offer more promotions, with the most common being low delivery fees, stamp cards and percentage off. Price and promotion strategies differed across cuisines and outlet types.

**Conclusions:**

Online menu item prices are relatively high, and promotions are widespread in the UK. Food outlets serving deprived areas often offer lower prices and more promotions. These targeted pricing and promotional strategies may influence purchasing behaviour and contribute to diet and health inequalities. Further research is needed to assess their impact on dietary behaviours and population health and guide policy interventions in the digital food environment.

Online food delivery services have become increasingly popular worldwide. The global online food delivery market was estimated to generate approximately one trillion US dollars in revenue in 2023, with around 40 billion in the UK^(1,2)^. These services, commonly provided by meal delivery apps (MDA), have extended the reach of traditional food outlets, allowing customers to order from the comfort of their homes or workplaces. However, this convenience comes at a cost: studies from various countries have consistently reported that the majority of food outlets, food offerings or food promoted on MDA are unhealthy^(3–7)^. This increased exposure to unhealthy options could lead to increased consumption of these foods, which presents a risk to public health^(8)^.

At the same time, however, MDA offer a unique opportunity to promote healthier diets in the digital food environment. By greatly expanding food access, these platforms can enable individuals living in ‘food deserts’ or ‘food swamps’ to obtain healthier food options through delivery^(9)^. Achieving this goal, however, will require thoughtful policy actions to reshape the current digital food environment, where unhealthy options predominate^(3–7,10)^. According to a recent WHO report, there have been no regulations specifically targeting MDA in the WHO European Region^(11)^. Most existing food and nutrition policies aimed at promoting a healthy food environment were introduced before this digital transformation and are not directly applicable to MDA^(11,12)^.

Policies could help leverage the potential of MDA to improve, rather than harm, population diet and health. A starting point could be adapting existing food and nutrition policies to the digital out-of-home food environment, including digital food marketing strategies^(13)^. For example, price promotions, special offers and the positioning of menu items can greatly influence consumer behaviour^(14,15)^. A randomised controlled trial found that prominently positioning lower-energy items on a simulated food delivery platform resulted in lower-energy purchases^(16)^. Consequently, the existing restrictions on the promotion of foods and drinks that are high in fat, salt, and sugar (HFSS) in England could be expanded to include out-of-home foods on online platforms, potentially reducing the overall energy intake^(17)^.

The first step in any policymaking effort, however, is to establish a baseline understanding of the current landscape, which is currently lacking for MDA. Existing literature on MDA has primarily focused on geographical access and healthiness of foods^(18)^. Other dimensions – such as price, promotion and placement, which, in addition to ‘product’ constitute the ‘marketing 4Ps’ – remain largely unexplored. Qualitative interviews with MDA users have shown that food affordability is a fundamental food purchasing consideration when ordering food delivery^(19)^. In supermarket settings, price promotions tend to increase consumer food and beverage purchases, with greater influence on the sales of unhealthy foods^(15)^.

To gain a comprehensive understanding, different aspects of the digital food environment should be considered collectively, rather than focusing solely on the availability and healthiness aspects. Menu item promotions offered could be used as powerful policy levers (e.g. restrictions on what items can be promoted on MDA) to improve the health and sustainability of food sold in the digital food environment.

The aim of this article was to first describe patterns of prices and promotions on a leading MDA, *JustEat*, in the UK. In 2023, JustEat generated over 1·3 billion euros in revenue across the UK and Ireland and represented over 40 % of the MDA market share in those countries^(20,21)^. Previous research has also shown that the availability and menu healthiness of out-of-home food outlets are socio-economically patterned in the UK, which caused a double burden of risk in deprived neighbourhoods^(22)^. As such, we also aimed to investigate whether menu item price and promotions offered by online food outlets were socio-economically patterned.

## Methods

**Figure 1.**
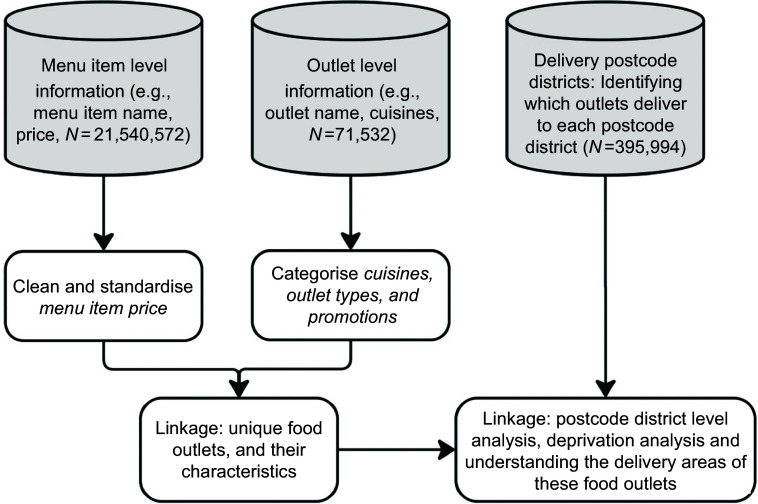
Flowchart of meal delivery app data linkage.

We collected data from all food outlets listed on *JustEat* across every postcode district in the UK in 2023. Using this dataset, we described the median price of menu items and the type of promotions available. We also sought to explore variations across different cuisine and outlet types. Thus, we explored these elements in relation to specific cuisines (e.g. ‘chicken dishes’, ‘pizza’) and outlet types (e.g. ‘large takeaway chains’, ‘grocery stores’). We further investigated the socio-economic patterns associated with these two aspects. General steps of data cleaning and linkage are shown in Figure 1.

### Menu and outlet data from *JustEat*

#### Data collection

We used an application programming interface to collect outlet and menu item-level information from *JustEat*, the leading MDA in the UK. In 2022, *JustEat* comprised 44·5 % of the market share, followed by *UberEats* at 28·3 % and *Deliveroo* at 27·3 %^(21)^.

The data collection was a two-step process. We first collected information on outlets that delivered to the centroid of each postcode district in the UK (step 1). A postcode district refers to the first part of a UK postcode, known as the outward code, which identifies a broader geographic area. For example, in the postcode CB2 1DQ, the district is ‘CB2’. In England and Wales, each postcode district has an average population of approximately 25 000 usual residents, according to the 2021 Census^(23)^. For each outlet, we collected the outlet name, ID, physical location, delivery postcode district, cuisine tags and promotions. This step was initially conducted during lunchtime on 29 September 2023 and repeated during dinnertime on 1 October 2023. For our analysis, we used the outlet data collected during dinner, as both datasets were highly similar, with 99·6 % of outlets being identical. Menus were scraped through the outlet’s URL, so the collection of menu data was also unaffected by whether the outlet was open at the time of scraping.

The second step involved collecting the menu information for each unique out-of-home food outlet identified in step 1. We collected menu item level information, including the menu item name, description, price and energy content (if available) on 1st October 2023. All data were collected and saved in JSON (i.e. a lightweight data-interchange format). For this study, we included only postcode districts that have at least one food outlet delivering to them in the UK (84·63 %). Postcode districts without delivery from any food outlets may include national parks or other remote areas.

#### Data processing

We processed the data following the steps below:

##### Price data

We defined a ‘menu item’ as any purchasing option listed on a menu, including all food and beverage items, as well as non-food and non-beverage options. For our price data analysis, we set the price to missing if it equalled zero (7·29 %). This could occur for several reasons: for instance, if an item was part of a meal deal, the meal deal options were recorded as individual item rows, with only one item assigned the full price and the remaining items listed at £0. A menu item not available at the time of collection also resulted in a base price of zero. We also observed extreme price outliers in our dataset, likely due to errors on the website (e.g. a bottle of wine for £21 474 836). To minimise the influence of these errors, we defined outliers as the top or bottom 0·05 % and set them as missing. 99·9 % of menu items fell within the price range of £0·20 and £66·90.

##### Cuisine classification

Online food outlets on *JustEat* had a total of 171 unique cuisine labels. We organised these labels into eleven cuisine categories: South Asian, Southeast & East Asian, Chicken Dishes, Kebabs, Burgers, Sandwich/Café/Bakery, Fish & Chips, Desserts, Pizza, Grocery and Other Cuisines. This categorisation was an adaptation of the ten-point classification system developed by Bishop et al.^(24)^, with the addition of a ‘Grocery’ category and the exclusion of ‘Multi Fast Food’. Detailed label-to-category mappings are available in online supplementary material, Supplemental Appendix File 1. Our cuisine analysis focused on non-grocery takeaways.

##### Food outlet type

We also classified online food outlets into three categories: grocery stores, major chain takeaways and independent/small-scale takeaways. This categorisation was based on the hypothesis that grocery stores differ from takeaway shops in terms of price promotions and their positioning on the home page, and major chains differ from independent takeaways in similar aspects. As major chains with 250 or more employees are required to display energy information on menus or at the point of sale, including on third-party aggregator platforms, we used percentage of items displaying energy information on *JustEat* as a proxy to identify chains^(25)^. This approach assumed full compliance with the energy labelling policy by all major chains, though research in other countries has shown inconsistent adherence to similar regulations^(26,27)^. We experimented with various percentage cut-offs – 25 %, 50 %, 75 % and 100 %. Notably, very few outlets displayed energy content for all items, as some items are exempt (e.g. alcoholic drinks) from the energy labelling policy. On the other hand, if the percentage of items displaying energy information was very low (e.g. 5 %), it could mean that the outlet had a few packaged items which had nutritional information, rather than being an indication of a chain takeaway. Ultimately, we settled on a 25 % threshold for menu items displaying energy content, with an exception for Tortilla, whose energy information became available upon clicking on item customisation. Thus, our classification criteria were as follows: grocery stores were those with a cuisine tag ‘grocery’, major chain takeaways as non-grocery stores with at least 25 % of menu items listing energy information and independent/small-scale takeaways as outlets not meeting the former criteria.

##### Promotions

Promotion types included price reductions (e.g. percentage off), stamp cards, free items, meal deal notifications, volume-based price promotions (e.g. buy one get one free) and low delivery fees. For ‘buy one get one free’ promotions, we included both standard and mixed-match variations. All other promotion types were coded as defined by *JustEat*. We binary coded the presence of each promotion type.

### Postcode district-level variables

#### Area deprivation and urban/rural status

The National Statistics Postcode Lookup for the UK, as of August 2023, was obtained from the Office for National Statistics and linked postcodes to various 2021 census geographies^(28)^. Multiple deprivation is a small-area measure of relative deprivation in the UK, combining factors such as employment, education and income^(29–31)^. We calculated the average deprivation ranks for each postcode district by averaging the ranks of all lower layer super output areas (LSOA) within or intersecting these districts. LSOA is a census geography unit, usually comprising 1000–3000 persons^(32)^. Ranks assigned a value of 0 were considered missing, as 0 was used to indicate missing data in the lookup. We then created deprivation rank quintiles separately for each country within the UK – England, Wales, Scotland and Northern Ireland – due to their different ranking systems.

Additionally, we addressed discrepancies between postcode districts in the National Statistics Postcode Lookup and those used in our data collection. For example, we adjusted ‘SW1Y’ (a postcode district used in the National Statistics Postcode Lookup) to ‘SW1’ (a postcode used in our data collection) and made similar adjustments for ‘WC1’, ‘EC1’, ‘EC2’, ‘EC4’, ‘WC2’, ‘EC3’ and ‘SW1’. If multiple postcode districts were present in the National Statistics Postcode Lookup, we calculated the average deprivation values for each postcode district included in our collection.

We categorised each postcode district as either ‘urban’ or ‘rural’ based on their intersecting and contained LSOA. If the number of urban LSOA was equal to or greater than the number of rural LSOA, the postcode district was classified as urban; otherwise, it was classified as rural.

### Statistical analysis

We calculated the median, maximum and minimum item prices for each outlet. The Kruskal–Wallis H test was used to test for differences in median item prices between different outlet types and outlets with different cuisine tags. To investigate the relationship between median menu item price and area deprivation, we conducted the analysis at the food outlet level. We focused our analysis on the delivery locations of online food outlets, rather than their physical locations, as these more accurately capture the population’s actual exposure to food outlets on MDA. Food outlets delivering to multiple postcode districts were counted as separate records, under the assumption that a larger delivery area indicated a greater presence. This approach yields estimates equivalent to weighting by the number of postcode districts an outlet delivers to.

The intra-class correlation (ICC) for median price at the postcode district level was very low (0·05), so we did not account for clustering at this level. Since the median price of menus at the food outlet level was normally distributed, we used generalised linear regression models to examine associations between median menu item prices and area deprivation. Models were adjusted for the urban/rural status of the delivery area (‘covariate’). We did not adjust for the total number of food outlets delivering to the postcode district, cuisine tags and whether the outlet was a chain. These factors were deemed mediators rather than confounders.

In our analysis of the relationship between the total number of promotions per outlet and area deprivation, the ICC was 0·13, suggesting some clustering at the postcode district level. To address this, we used linear mixed models with random intercepts. These models showed better performance compared with Poisson mixed models, as evidenced by a lower Akaike Information Criterion. Therefore, we modelled the number of promotions as a continuous variable rather than as count data. As a first step, we modelled the *total number* of promotions at the postcode district level by IMD quintiles. This provided a descriptive, absolute count of promotions for food outlets delivered to postcode districts within each IMD quintile. We then modelled the number of promotions *per outlet* by IMD quintiles. This gave an understanding of the average promotions for each food outlet within each IMD quintile.

We further stratified the analysis by type of promotion. We calculated the ICC values for each promotion type at the postcode district level. Only the low delivery fee (ICC = 0·43) and buy one get one free (ICC = 0·16) promotions had an ICC greater than 0·06. For these two types of promotions, we used logistic mixed regression models with random intercepts to account for clustering. For other promotion types, we used generalised logistic regression models. We adjusted for urban/rural status for each model. The marginal predictive values from adjusted models were the population marginal effects at mean. We processed and cleaned the data using Python (version 3.8.2) and conducted statistical analyses using R (version 4.3.0).

## Results

### Characteristics of online food outlets

Our dataset comprised 21 540 572 menu items from 71 532 unique food outlets across the UK. As shown in Table 1, out of the 71 532 unique food outlets, 71·83 % were small-scale takeaways, 21·65 % were major chains and the remaining 8·31 % were grocery stores. Out of the 65 635 outlets where ‘grocery’ was not the first listed tag, the top three cuisine tags were sandwich/café/bakery (17·51 %), pizza (16·86 %) and south Asian (13·23 %). A total of 395 994 records were identified as delivering to 2521 postcode districts (3118 total postcode districts in the UK). Across all outlets, the median number of menu items was 160 (IQR: 84–282). Grocery stores had a substantially higher number of menu items per outlet (median: 1190; IQR: 437–2612) compared with other types of food outlets. The median number of food outlets accessible via *JustEat* was 69 for postcode districts with delivery access (IQR = 14–225).

**Table 1.** Unique food outlets by type and cuisine tags

Labels	Unique food outlets		Number of menu items for each unique food outlet	
	*n*	%	Median	IQR
Type of outlets				
Small scale takeaways	51 380	71·83 %	133	74–230
Major chains	14 209	21·65 %	212	104–354
Grocery stores	5943	8·31 %	1190	437–2612
Cuisine tags for outlets where ‘grocery’ is not the first listed tag				
Sandwich/cafe/bakery	11 491	17·51 %	195	95–285
Pizza	11 063	16·86 %	209	116–352
South Asian	8682	13·23 %	224	137–307
Southeast and East Asian	6902	10·52 %	193	95–267
Burgers	6311	9·62 %	76	51–123
Chicken dishes	5725	8·72 %	109	76–164
Kebabs	4423	6·74 %	144	89–223
Not classified	3910	5·96 %	65	41–93
Desserts	3698	5·63 %	107	51–178
Fish and chips	3430	5·23 %	113	77–174

### Menu item prices

Overall, the median value for *median menu item* price at the outlet level was £6·25 (IQR = £4·09–£7·99). The median *maximum menu item price* was £24·99 (IQR = £14·99–£39·99), and the median *minimum menu price* was £0·80 (IQR = £0·50–£1·20).

#### Median menu item price by outlet type and cuisine types

**Figure 2.**
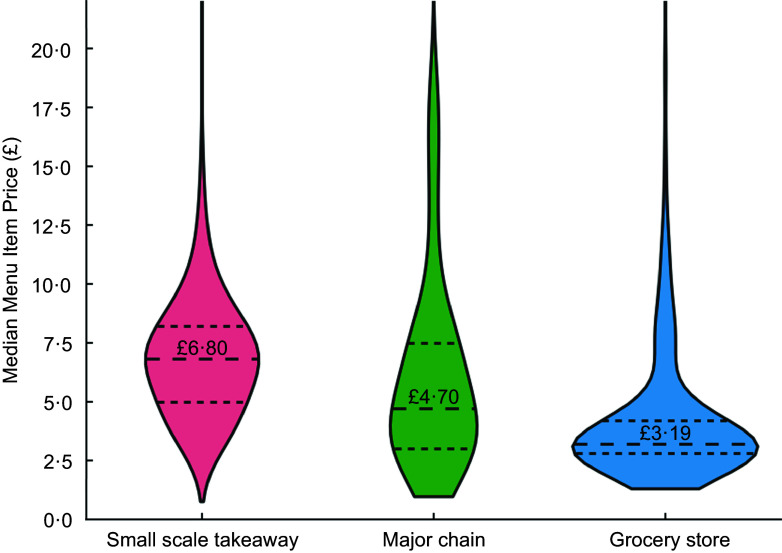
Median menu item price distribution of outlets available on *JustEat*, by type of food outlets.

The distribution of median menu item price by type of food outlet is shown in Figure 2. The median value of *median menu item price* was the highest for small-scale takeaways at £6·80 (IQR = £5·00–£8·20), followed by major chains at £4·70 (IQR = £3·00–£7·49) and grocery stores at £3·19 (IQR = £2·79–£4·19). There was a statistically significant difference in median item price between different types of food outlets (*P* < 0·001).

**Figure 3.**
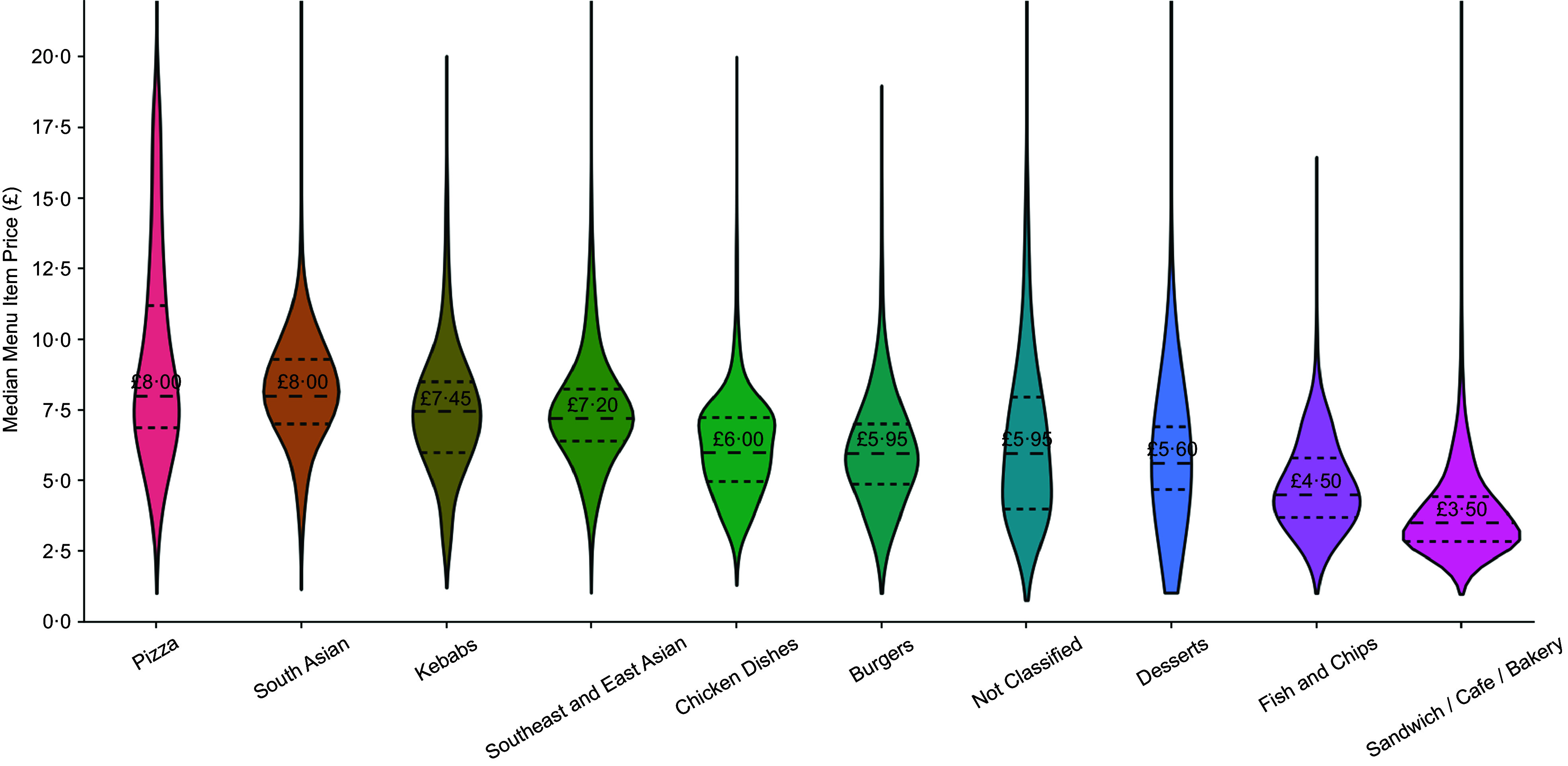
Median menu item price distribution of outlets available on *JustEat*, by cuisine tag.

In terms of cuisine tags for outlets where ‘grocery’ is not the first listed tag, the data showed notable variations in median menu item prices (Figure 3, *P* < 0·001). Pizzas and South Asian cuisines had the highest median prices at £8·00 and £7·00, respectively, while Sandwiches/Cafés/Bakery had the lowest at £3·50. The distribution of prices varied across cuisine types, with pizzas showing a wider range, indicating a wider variety of offerings at different price points. In contrast, the pricing for items offered by sandwich/café/bakery outlets was more consistent, likely reflecting the narrower range of products, such as coffees, typically offered at similar prices.

#### Menu item price and area deprivation

**Figure 4.**
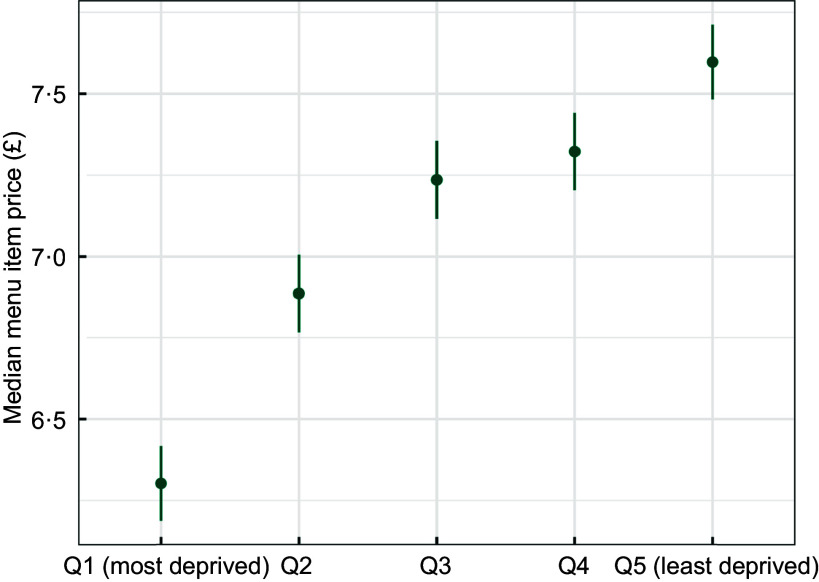
Median menu item price, by area deprivation (adjusted for urban/rural status).

There was a clear gradient in the median menu item price by area deprivation (*P* < 0·001). As shown in Figure 4, in the most deprived postcode districts, the mean *median menu item price* was £6·30 (mean predicted values from the adjusted model, 95 % CI = 6·19, 6·42), while in the least deprived it was £7·60 (95 % CI = 7·48, 7·71). The unadjusted crude *median menu item price* by deprivation quintile followed the same pattern (not shown, due to substantial overlap). The most deprived postcode districts had the lowest average median menu item price for food outlets delivering there (£6·42, 95 % CI = 6·40, 6·44), and the least deprived areas had the highest (£7·31, 95 % CI = 7·27, 7·34).

### Promotions

#### Different types of promotions

Online food outlets on the MDA offered six types of product promotions to customers: percentage off, stamp cards, free items, meal deal notifications, buy one get one free and low delivery fees for delivery services. Table 2 presents the definition, examples and prevalence of each promotion type. Across all unique food outlets on *JustEat*, the most prevalent type of promotion was a low delivery fee (35·44 %), followed by stamp card (26·66 %), percentage off (18·26 %), meal deal notifications (10·29 %), free items (6·49 %) and buy one get one free (0·06 %). However, when we calculated the percentage of food outlets offering each promotion at the *postcode district level*, the two types of promotions with the highest average percentages were stamp card (29·41 %, IQR = 21·37–35·99 %) and percentage off (19·78 %, IQR = 12·50–27·27 %). The percentage for low delivery fee at the postcode district level was observed to be lower, at 10·29 % (IQR = 0–21·56 %). This may be attributed to the fact that promotions offering low delivery fees were more concentrated in areas with a greater number of online out-of-home food outlets. As a result, while the absolute number of such promotions was high, their average percentage at the postcode district level remained relatively low.

**Table 2.** Types of promotions offered by online food outlets

Type of promotions	Definitions	Examples	Percentage of *unique online food outlets offering* *promotion*	Percentage of *delivery outlets offering promotion (postcode district level)*
Percentage off (‘price reduction’)	A percentage reduction on the total order amount.	•‘10 % off when you spend £25’ •‘10 % off your order today’ •‘20 % off when you spend £20 before 23.59’	18·26 %	19·78 %
Stamp card	A loyalty programme where customers earn stamps for each purchase. Collecting five stamps from the same restaurant unlocks a discount on the sixth order, with each stamp valued at 10 % of the order.	If the first five orders from a restaurant offering a stamp card are valued at £40, £50, £45, £45 and £55, then the total discount applied to the sixth order is £23 (10 % of the total five orders).	26·66 %	29·41 %
Free item	After a qualifying purchase, a free item can be added to your order.	•A free hash brown from McDonalds •A free ginger shot from Joe & the Juice	6·49 %	7·14 %
Meal deal notification	Meal deal notification appears on the home page for the food outlet once you enter your address.	•‘Save up to 20 % when you purchase our Value Bundles’ •‘Save with an exclusive meal deal’	10·29 %	6·25 %
Buy one get one free (‘volume based price promotions’)	Buy one get one free, including buy one get one free mix and match.	•Promotions offer *JustEat* customers one free Ben & Jerry’s 465 ml Ice Cream (of same flavour) when they order a Ben & Jerry’s 465 ml Ice Cream	0·06 %	0·00 %
Low delivery fee	‘Low delivery fee’ listed as one of the labels on the home page – usually free delivery.	•‘Delivery for free, no minimal order’	35·44 %	10·29 %

#### Promotions by type of food outlets and cuisine tags

Among all unique online food outlets, 65·96 % offered some type of promotion. Specifically, 82·79 % of grocery stores, 69·84 % of major chains and 62·94 % of independent/small takeaways had at least one promotion. When stratified by cuisine tags, the percentage of unique online outlets offering promotions ranged from 51·45 % (Fish and Chips) to 77·48 % (Chicken dishes).

**Figure 5.**
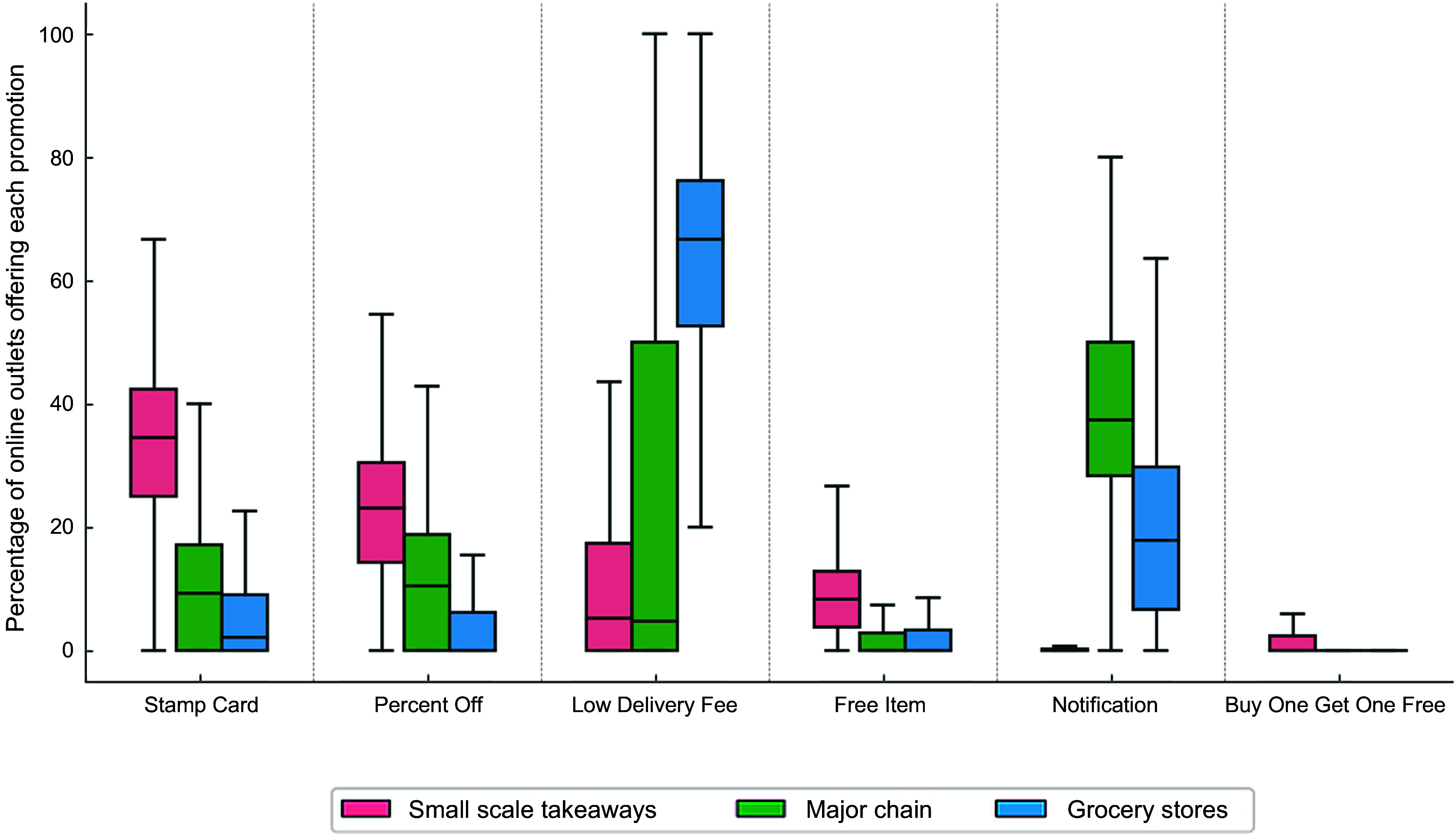
Percentage of online outlets offering each promotion (postcode district level), by type of outlet.

The median number of promotions was 1 for all types of food outlets. However, types of promotions differ across different food outlets on the postcode district level. As shown in Figure 5, the most frequently used promotional tactic in grocery stores was the low delivery fee (median percentage of grocery stores offering low delivery fee: 66·67 %, IQR = 52·63–76·10 %), while in major chains, it was meal deal notification (37·50 %, IQR = 28·39–50·00 %), and in small scale takeaways, it was the stamp card (34·48 %, IQR = 25·00–42·28 %). The percentage off promotion appeared to be most commonly used in small-scale takeaways (23·08 %, IQR = 14·29–30·56 %), followed by major chains (10·54 %, IQR = 0·00–18·75 %), and was rarely used in grocery stores (0·00 %, IQR = 0·00–6·25 %)

In terms of cuisine tags for non-grocery takeaways, desserts (median = 1·06, IQR = 0·78–1·57), pizzas (1·00, IQR = 0·77–1·36) and chicken dishes (1·00, IQR = 0·75–1·50) had the highest average number of promotions per outlet. Sandwich/café/bakery (0·67, IQR = 0·43–1·20) and Southeast and East Asian (0·50, IQR = 0·20–0·94) had the lowest number of promotions per outlet. They also used different types of promotions: for example, low delivery fees were particularly common in Sandwich/Café/Bakery outlets (44·95 %) and chicken dishes (50·45 %). Stamp card was most popular among desserts (52·55 %), burgers (39·72 %), chicken dishes (38·16 %) and pizza outlets (33·89 %), but less common in Southeast and East Asian outlets (26·86 %).

#### Promotions and area deprivation

##### Total number of promotions

**Figure 6.**
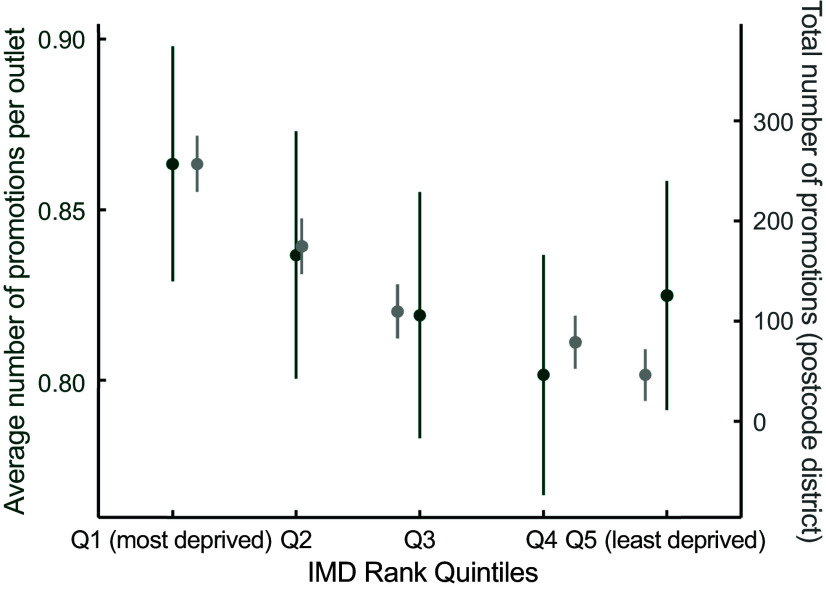
Total and average number of promotions, by IMD rank quintiles.

The *average number of promotions per outlet*, by IMD quintiles, showed a decreasing trend (Figure 6, *P* < 0·001), followed by a slight increase for the least deprived postcode districts compared with the second least deprived. The number of promotions per outlet was 0·86 (95 % CI = 0·83, 0·90) for the most deprived, 0·84 (95 % CI = 0·80, 0·87) for Q2, 0·82 (95 % CI = 0·78, 0·76) for Q3, 0·80 (95 % CI = 0·77, 0·84) for Q4 and 0·82 (95 % CI = 0·79, 0·86) for the least deprived. In terms of the *total number of promotions at the postcode district level*, there was a clearer gradient. The most deprived postcode districts had the highest number of promotions (256·91, 95 % CI = 228·79, 285·03), followed by Q2 (174·71, 95 % CI = 146·79, 202·64), Q3 (109·63, 95 % CI = 82·50, 136·75), Q4 (78·93, 95 % CI = 52·38, 105·49) and the least deprived, which had the lowest (46·36, 95 % CI = 20·49, 72·23).

##### Different types of promotions

**Figure 7.**
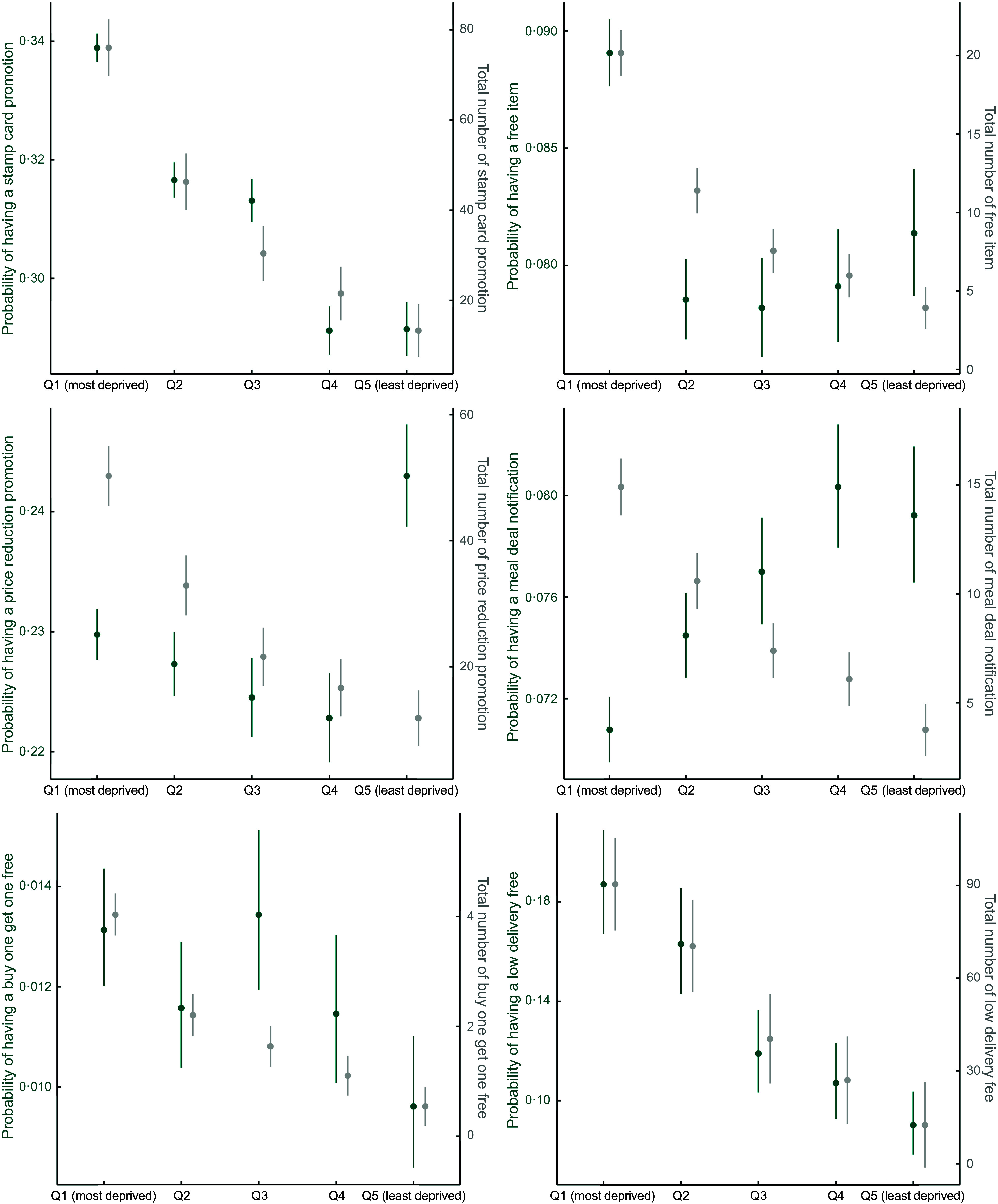
Total and average number of promotions by deprivation, stratified by type of promotion.

Similar to the total number of promotions available at the postcode level, the trend persisted when stratified by type of promotion (Figure 7): there were generally more promotions for food outlets delivering to more deprived postcode districts. This could have been driven by the fact that there were more out-of-home food outlets deliver to these areas, which increased the total number of promotions^(33)^.

In terms of the probability of having a promotion, stamp card promotions, free item promotions and low delivery fees were the most commonly used tactics by online food outlets delivering to the most deprived postcode districts. Conversely, percentage-off promotions and meal deal notifications were more commonly used in less deprived postcode districts. There was no clear pattern for the buy one get one free promotion by deprivation level, but the third deprivation quintile had the highest probability of featuring such a promotion.

## Discussion

### Summary of findings

This study examined menu item prices and promotions offered by food outlets on *JustEat*, a leading online food delivery platform in the UK. We found that the median menu item price was £6·25. There were six types of promotions available: low delivery fees, stamp cards, percentage off, meal deal notifications, free items and buy one get one free offer. More than 65 % of online food outlets offered promotions, with the most common being low delivery fees, stamp cards and price reduction. We also found that food outlets delivering to more deprived areas tended to offer lower-priced items with more promotions. The price and type of promotions varied by cuisine and food outlet type. For example, item prices were generally higher in pizza takeaways and lower in sandwich/café/bakeries. Low delivery fee promotions were commonly used by grocery stores, while meal deal notifications were more popular among major chain takeaways.

### Research interpretation

In the UK, the average weekly household food expenditure was £41·11 per person in 2022, including food prepared out-of-home^(34)^. The median menu item price was £6·25 for food available on MDA, which equates to 15 % of the weekly food budget. Given that the average expenditure on food and drink *eaten out* was only £8·94, the median item price would consume approximately 70 % of that budget^(34)^. Factoring in delivery and service fees, a single order from these apps can take up an even greater share of the weekly food expenditure. This could make ordering from MDA prohibitively expensive for individuals of lower socio-economic statuses. This is in line with the research finding that individuals who use MDA are more likely to have more education and income^(35,36)^. Consequently, we found that menu items offered by food outlets delivering to more deprived areas were priced lower, possibly reflecting the customer demand and economic reality of these areas. This also mirrors the trend observed in brick-and-mortar stores, where there are more food outlets in more deprived areas^(37)^. Lower prices may also lead to the use of lower-quality ingredients, which could result in less healthy food options in more deprived neighbourhoods^(22)^.

We also found that the median menu item price varied by type of food outlets and cuisine types, which could be influenced by the nature of the items sold in each type of food outlet. For instance, grocery stores often offer a variety of small, lower-priced items. Many chains on *JustEat*, such as Costa and Caffe Nero, are sandwich/café/bakery outlets that typically offer a range of lower-priced beverages. In contrast, median menu item prices tend to be higher in pizza restaurants, likely due to the fact that many pizza items are intended for sharing.

A narrative review published in 2020 highlights the lack of information on marketing strategies used by MDA^(38)^. In this study, we identified six types of promotion strategies: low delivery fees, stamp cards, price reductions, meal deal notifications, free items and buy one get one free offers. Among these, a unique type of promotion for online food delivery services, compared with other physical food services, is the offer of low delivery fees. In online retail settings, researchers have found that customers place the greatest importance on the delivery fee when making a purchase decision, more so than on other factors related to delivery such as delivery speed and time slot^(39)^. Online grocery stores on MDA, which often adopt this strategy, might attract and retain customers by offering lower delivery fees. However, despite being viewed as healthier, online grocery stores might not always offer healthy choices on MDA. They focus on foods high in fat, sugar and salt, as well as alcohol and tobacco products, which may lead to negative health implications^(40)^.

In this study, we identified stamp cards – a customer loyalty program – as a widely used promotional strategy. Compared with traditional physical stamp cards, digital versions of MDA provide a more efficient way to monitor customer purchases and spending. Such programmes are effective in the marketing domain, supported by the goal gradient hypothesis which suggests that individuals increase their effort as they approach a reward^(41)^. We also found that small, independent takeaways commonly use these promotions, likely to build loyalty due to their weaker brand recognition compared with major chains like McDonald’s. However, stamp card promotions raise public health concerns by likely accelerating repeat out-of-home food purchases. These promotions are particularly prevalent in independent takeaways, which tend to serve food that contains high levels of energy, fat and salt^(42,43)^.

Along with free item promotions (extra-product price promotions), these three types of promotions are also more likely to be found in the most deprived neighbourhoods. Conversely, percentage off and meal deal notifications are more likely to be found in less deprived neighbourhoods. The type of promotions offered across neighbourhoods within various deprivation quintiles may be explained by the different types of food outlets in these areas. The higher food prices in these areas also enable percentage off promotions, as their profit margins are likely to be larger. The use of different promotional tactics based on the level of area deprivation – such as offering percentage off for food outlets delivering to less deprived areas and free items for food outlets delivering to more deprived areas – indicates a targeted approach that might encourage consumption patterns influenced more by promotions. Overall, there are more total promotions and a higher average number of promotions for food outlets delivering to deprived areas. This is likely due to the greater number of online out-of-home food outlets in these areas, leading to increased competition and a greater need to offer promotions^(33)^.

While sales promotions are shown to increase sales over the short term, they may not necessarily lead to changes in food consumption patterns^(44)^. For example, in supermarket settings, consumers may buy more food as a result (‘stockpiling’) but may consume it at a regular or faster speed. More research is needed to understand how each type of promotion will influence the out-of-home purchasing and consumption behaviour.

The factors that determine an outlet’s pricing and promotional strategies remain unclear and warrant further investigation. It is important to explore whether these practices are shaped by customer demand, retailer and MDA marketing strategies, and/or internal platform policies and understand the mechanisms and rationale behind them. As a few powerful MDA grow increasingly dominant, there is concern that profit may be prioritised over public health. For example, recent research on UberEats has documented targeted marketing to families and children and other practices that consolidate market and political power, raising important questions about the broader role of MDA in shaping food environments^(45)^. Future research should also examine the potential health impacts of different pricing and promotion strategies to better inform policy development in the digital food environment.

### Strength and limitations

The study leverages a dataset from *JustEat* with over 21 million menu items from over 70 000 food outlets, which offers a comprehensive analysis of item prices and promotions offered online in the UK. Previous research on MDA has primarily focused on the product aspect of the 4Ps, such as the healthiness of items offered or promoted, as well as the accessibility of online food outlets^(3,5,6,46,47)^. To our knowledge, this is the first study to examine item price and promotions—two other key elements of the 4Ps – on an MDA on a national scale.

However, this study is not without its limitations. In this study, we exclusively used data from *JustEat*, without including data from other MDA such as *Deliveroo* and *UberEats*, which accounted for approximately 55 % of the food delivery service market share in 2023^(21)^. A previous study in the UK exploring the relationship between area deprivation and availability of online outlets found varying associations by platform, which suggests that it might be relevant to combine these data sources^(48)^. However, due to resource constraints, we were unable to link menu records from all three platforms. Additionally, we used the presence of energy labels to identify large chains as these are required by law in England to display energy labels at the point of choice. Research from Australia and Canada has shown that there is inconsistent adherence to similar energy labelling regulations on MDA there^(26,27)^. We are not aware of similar research from the UK. However, if adherence is also inconsistent in the UK, this may have led to the misclassification of some outlets.

For the analysis of price, we only focused on menu item prices and did not assess delivery fees, which could vary based on the customer’s ordering location. However, delivery fees may be an important factor in food ordering decisions. Although customers are generally willing to pay a premium for delivery, if the fee is excessively high, they may consider alternatives^(49)^. For the analysis of promotions offered by food outlets, we also only included promotions shown on MDA websites. Individualised promotional offers, such as ‘50 % off your next order’, made available in personal accounts and also notified through email, were not included. However, we do not have individual-level promotion data. Furthermore, our analysis could not investigate the placement of food outlets (i.e. ordering of outlets in the display) due to the user-specific display presented by MDA. This placement likely incorporates both user location and preferences.

Moreover, in this study, we were unable to conduct an analysis by menu item category (e.g. beverages, burgers, pizzas) due to the significant resources required to categorise all twenty-one million menu items. We also did not exclude non-food and non-beverage items such as household goods, toiletries, healthcare products and tobacco, which are typically sold by grocery stores. While sub-analyses by outlet type may partially capture this, it would be useful to explore how these items differ from food and beverage offerings. Future studies could employ machine learning or deep learning techniques to categorise these data and provide further insights at the menu item category level. For example, they could explore whether less healthy menu items are more likely to be promoted at the top within an outlet.

Lastly, we did not examine the impacts of price and promotions on food delivery purchasing, consumption, diet or health. This analysis remains descriptive and focused on understanding specific aspects of the digital retail environment. Further research is needed to establish whether policy intervention is warranted and, if so, what form it should take.

### Conclusion

In this study, we characterised the pricing and promotional strategies on meal delivery platforms. Over 65 % of online outlets offer some form of promotion. We found that menu item prices are lower and more promotions are available for outlets delivering to areas with greater deprivation. Further research is needed to understand how these factors influence dietary behaviour and health outcomes. Policy efforts may be necessary to ensure equitable pricing and promotion practices on these platforms.

## Supporting information

Huang et al. supplementary material Huang et al. supplementary material

## Supplementary material

For supplementary material accompanying this paper visit https://doi.org/10.1017/S1368980025100529
