# Supplementary material for: Menu item prices and promotions offered on a meal delivery app in the UK and their socio-economic patterns
Source: Public Health Nutr. 2025 Jun 13;28(1):e110. doi: 10.1017/S1368980025100529 (PMC12305391; doi:10.1017/S1368980025100529)
Supplement: Huang et al. supplementary material [file S1368980025100529sup001.docx]

# Supplementary Appendix File 1

**Table S1:** Cuisine to category mapping dictionary

| Category | Cuisines |
| --- | --- |
| South Asian | Indian, Curry, Bangladeshi, Nepalese, Pakistani, South Indian, Sri-lankan/Sri Lankan, Balti, Punjabi, Biryani, Indonesian |
| Southeast & East Asian | Asian, Chinese, Oriental, Thai, Japanese, Noodles, Pan-Asian, Poke, Sushi, Vietnamese, Malaysian, Korean, Bubble Tea, Cantonese, Dim Sum, Singapore, Filipino, Taiwanese, Mongolian |
| Chicken Dishes | Chicken, Peri Peri |
| Kebabs | Kebab, Turkish |
| Burgers | Burgers, American, Gourmet Burgers |
| Sandwich / Cafe / Bakery | Breakfast, Sandwiches, Café, Deli, Wraps, Salads, Paninis, Bagels, Baguettes, Bakery, Coffee, Doughnuts, Café |
| Fish and Chips | Fish & Chips |
| Desserts | Desserts, Ice Cream, Milkshakes, Cakes, Waffles, Crepes |
| Pizza | Pizza, Italian, Italian Pizza |
| Promotions | Deals, Low Delivery Fee, Collect stamps, Freebies |
| Grocery | Groceries, Supermarkets, Alcohol |
| Not Classified | Grill, Caribbean, British, English, Mediterranean, Lebanese, Fast Food, Halal, Mexican, Vegan, Jamaican, Vegetarian, Healthy, Pasta, Lunch, African, Greek, Middle Eastern, Persian, European, Gourmet, BBQ, Steak, Drinks, Afghan, Fusion, Arabic, Spanish, Nigerian, Seafood, Street Food, Burritos, Iranian, French, Dinner, Portuguese, Smoothies, Tex Mex, Sweets, Tapas, Moroccan, English Breakfast, Jerk, Roast Dinners, Romanian, None, Gluten Free, Parmesans, Polish, Brazilian food, Brunch, South American, Eastern European, Indo-Chinese Fusion, Pub Food, Russian, Ethiopian, Kurdish, Continental, Pancakes, Latin American, Hot Dogs, Syrian, North African, Pies, Organic, Kosher, Hungarian, Frozen Yogurt, Peruvian, South African, German, Colombian, Authentic Pizza, Bulgarian, Scottish, West African, Retro Sweets, Alcohol, Argentinian, Soup, Egyptian, Ukrainian, Rotisserie, Danish, Sizzlers, NEW, Subways, Low-Carb, Belgian Waffles, Best Bites, Tex-Mex, Ghanaian, Iraqi, Salt & Pepper, Carvery, Pies , Grains & Cereals, Mocktails, Venezuelan, all night alcohol, Georgian, Smoke House, Cuban, Pie and Mash, Trinidadian, *New*, Mauritian, Pick n Mix, Local Legends |
